# Direct conversion of fibroblasts into urothelial cells that may be recruited to regenerating mucosa of injured urinary bladder

**DOI:** 10.1038/s41598-019-50388-6

**Published:** 2019-09-25

**Authors:** Yuta Inoue, Tsunao Kishida, Shin-ichiro Kotani, Mika Akiyoshi, Hideto Taga, Makoto Seki, Osamu Ukimura, Osam Mazda

**Affiliations:** 1 Department of Immunology, Kyoto Prefecture University of Medicine, Kamigyo-ku, Kyoto, 602-8566 Japan; 2 Department of Urology, Kyoto Prefecture University of Medicine, Kamigyo-ku, Kyoto, 602-8566 Japan; 3 CellAxia Inc. 1-10-9-6F Nihonbashi Horidome-cho, Chuo-ku, Tokyo, 103-0012 Japan

**Keywords:** Bladder, Bladder disease, Transdifferentiation, Reprogramming, Regenerative medicine

## Abstract

Urothelial cells play essential roles in protection of urine exudation and bacterial invasion at the urothelial mucosa, so that defect or damage of urothelial cells associated with urinary tract diseases may cause serious problems. If a sufficient number of functional urothelial cells are prepared in culture and transplanted into the damaged urothelial lesions, such technology may provide beneficial effects to patients with diseases of the urinary tract. Here we found that human adult dermal fibroblasts were converted into urothelial cells by transducing genes for four transcription factors, FOXA1, TP63, MYCL and KLF4 (FTLK). The directly converted urothelial cells (dUCs) formed cobblestone-like colonies and expressed urothelium-specific markers. dUCs were successfully expanded and enriched after serial passages using a specific medium that we optimized for the cells. The passaged dUCs showed similar genome-wide gene expression profiles to normal urothelial cells and had a barrier function. The FTLK-transduced fibroblasts were also converted into urothelial cells *in vivo* and recruited to the regenerating urothelial tissue after they were transplanted into the bladder of mice with interstitial cystitis. Our technology may provide a promising solution for a number of patients with urinary tract disorders.

## Introduction

Urothelial cells form urothelium on the surface of the urinary tract that is composed of renal pelvis, ureter, urinary bladder and proximal urethra. They expand and contract depending on the urine storage keeping a strong barrier against urine exudation and bacterial invasion. Some pathological situations of urinary tract such as bladder cancer, neurogenic bladder, congenital anomalies, bladder injury, interstitial cystitis, contracted bladder, are associated with loss of urothelial cells and/or of their functions. Damaged urothelium in patients could be treated by transplantation of autologous intestine or colon tissue segments, but these gastrointestinal tissues easily resorb urine, causing many problems including cancer formation^1^, metabolic acidosis, infection, stone formation, etc^2^.

In this context, a lot of efforts have been devoted to search for another source of the cells applicable to transplantation instead of gastrointestinal tissue segments. Atala *et al*. reported autologous transplantation of a bladder tissue that had been expanded in culture with a collagen-polyglycolic acid scaffold^3^. They achieved sufficient results for short-terms after the transplantation, but this procedure has not been widely used because the autologous bladder tissue may become dysfunctional after long-term follow-up. Other researchers induced urothelial cells from non-urothelial immature stem cells. Ning *et al*. cocultured human fetal bone-marrow-derived mesenchymal stem cells with patients’ urothelial cells^4^, while Shi *et al*. cultured human adipose-derived stem cells with conditioned medium of urothelial cells^5^. But these cells were hardly applicable to clinical use, because the procedures required autologous urothelial cells. Two reports showed generation of urothelial cells from human ES cells and induced pluripotent stem (iPS) cells^6,7^. However, transplantation of cells derived from human pluripotent stem cells could cause teratoma formation.

We considered that it might be promising if urothelial cells were generated by the direct conversion (direct reprogramming) technology that induces differentiated tissue cells from another somatic cell lineage without passing through a pluripotent state. Direct conversion can be achieved by transducing a set of genes encoding transcriptional factors that play crucial roles in the development of the target cell lineage. Hepatocytes^8^, cardiomyocytes^9^, neurons^10^, were induced from different lineage cells like fibroblasts by such procedures. An addition of genes that promote reprogramming of somatic cells into iPS cells may promote direct conversion of some cell types. In this context, POU5F1, KLF4 and MYC played crucial roles in directly converting fibroblasts into neural stem cells^11^ and chondrogenic cells^12^. We previously reported direct conversion from human fibroblasts into osteoblasts^13^, brown adipocytes^14^, Schwann cells^15^ and myoblasts^16^.

In this study, we aimed at directly converting human fibroblasts into urothelial cells by introducing some transcription factor genes. We established a procedure of the conversion, and analyzed phenotypes and functions of the resultant directly converted urothelial cells (dUCs).

## Results

### Adult human dermal fibroblasts were induced to show urothelial cell-like phenotypes by defined factors

**Figure 1:**
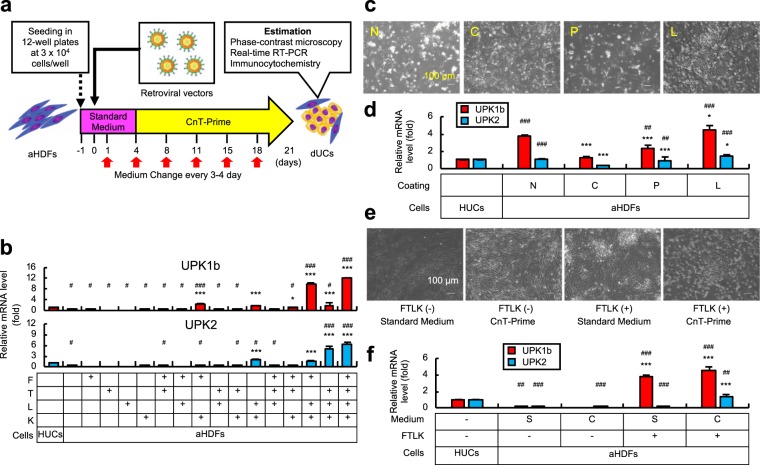
Exploration of a procedure to induce urothelial cell-like phenotypes in aHDFs. (**a**) Cell conversion procedures. (**b**) aHDFs were seeded in laminin-coated 12-well plates. On the next day (day 0), retroviral vectors containing FOXA1 (F), TP63 (T), MYCL (L) and KLF4 (K) genes were transduced into the cells as indicated by “+”. Cells were cultured in Standard Medium (days 1 to 3) and CnT-Prime (days 4 to 21). RNA was extracted from the cells and subjected to real-time RT-PCR to evaluate mRNA levels for the UPK1b and UPK2 genes. ^*^P < 0.05 and ^***^P < 0.001 vs. non-transduced aHDFs. ^#^P < 0.05 and ^###^P < 0.001 vs. HUCs. (**c**,**d**) aHDFs were seeded in non-coated (N) culture plates, or culture plates coated with collagen (C), poly-L-lysine (P) or laminin (L). After transduction with FTLK retroviral vectors (day 0), cells were cultured as in (**a**), and confocal microscopic imaging (**c**) and real-time RT-PCR analysis (**d**) were performed on day 21. ^*^P < 0.05 and ^***^P < 0.001 vs. N. ^##^P < 0.01 and ^###^P < 0.001 vs. HUCs. (**e,f**) aHDFs were seeded in laminin-coated plates, infected with or without FTLK retroviral vectors, and cultured for days 1 to 3 in Standard Medium. Cells were then cultured in Standard Medium (S) or CnT-Prime (C) until day 21, when confocal microscopic imaging (**e**) and real-time RT-PCR analysis (**f**) were performed. ^***^P < 0.001 vs. non-transduced aHDFs cultured in Standard Medium. ^##^P < 0.01 and ^###^P < 0.001 vs. HUCs. Values are mean +/− SD (n = 3).

We first focused on three transcriptional factors (TFs). FOXA1 (F) and IRF1 (I) are key TFs in the urothelial development^17^, while TP63 (T) is an epithelial stem cell-related TF expressed in urothelial stem cells^18^. We also added SHH (H) that is expressed in urothelial basal cells and mediates important signals for regenerative proliferation of epithelial cells in bladder^19^. Adult human dermal fibroblasts (aHDFs) were transduced with various combinations of these genes via retroviral vectors and cultured for 21 days in the CnT-Prime medium that we considered adequate to maintain urothelial cells including immature urothelial progenitors^20^ (Fig. 1a). It was found that FIT (FOXA1, IRF1 and TP63) and FIH (FOXA1, IRF1 and SHH) induced only a low level of expression of uroplakin 1b (UPK1b) that is a typical urothelial developmental marker^21^ (Supplementary Fig. 1A), and neither FIT- nor FIH- transduced cells formed colonies with epithelium-like appearance (Supplementary Fig. 1B). Then we added the genes for three reprogramming factors, POU5F1 (P), KLF4 (K) and MYCL (L). Some combinations of urothelium-related genes and reprogramming factors induced high levels of UPK1b expression, and it was suggested that FOXA1 should be included in such combinations (Supplementary Fig. 2). Next, requirement of I, T and H genes was assessed, and it was found that T but not I or H enhanced formation of epithelial colonies (Supplementary Fig. 3A,B). Then we determined requirement of P, K and L by combining them with FT (FOXA1 and TP63). FTLK (FOXA1, TP63, MYCL and KLF4) strongly induced expression of UPK1b mRNA and generation of epithelial colonies (Supplementary Fig. 4A,B). Moreover, FTLK-transduced cells expressed uroplakin 2 (UPK2) mRNA that is another urothelium-specific marker^22^ (Supplementary Fig. 4A).

We tested whether FTLK was necessary and sufficient for induction of UPK1b and UPK2 expression. The mRNA level for UPK1b was elevated in the FLK (FOXA1, MYCL and KLF4) transduced cells, while TLK (TP63, MYCL and KLF4) induced expression of UPK2 mRNA at a high level (Fig. 1b). FTLK most strongly induced both UPK1b and UPK2 mRNA (Fig. 1b). Taken together, we concluded that FTLK was the optimal gene combination to induce urothelial phenotype in aHDFs.

We examined how the coating of the culture plates would affect the efficiency of the cell phenotype conversion. Culture plates were coated with collagen, poly-L-lysine or laminin before seeding of aHDFs. Epithelial colonies were formed only in laminin-coated plates (Fig. 1c), in which the cells expressed both UPK1b and UPK2 mRNA at the highest levels (Fig. 1d).

FTLK-transduced cells and non-transduced cells were cultured in various culture media. As shown in Fig. 1e,f and Supplementary Fig. 5, FTLK-transduced cells formed most efficiently epithelial colonies, and significantly expressed UPK1b and UPK2 mRNA, in the CnT-Prime but not in the Standard Medium. The “Uromedium” that was reportedly suitable for urothelial cells^6^ supported not only formation of epithelial colonies but also proliferation of fibroblast-like cells. The results suggested superiority of the CnT-Prime for the urothelial-like cell type change. In contrast, the CnT-Prime was not sufficient for the non-transduced cells to be converted into urothelial cells.

### Characterization of the directly converted urothelial cells (dUCs)

**Figure 2:**
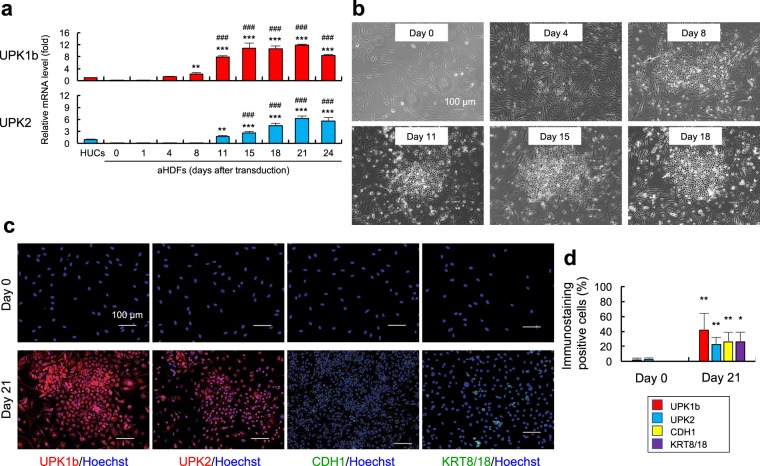
FTLK-transduced cells showed urothelial cell-like phenotypes. aHDFs were seeded in laminin-coated 12-well plates, transduced with FTLK, and cultured in Standard Medium (days 1 to 3) and CnT-Prime (days 4 to 21). (**a**) On the indicated days, RNA was extracted from the cells and subjected to real-time RT-PCR to evaluate mRNA levels for the UPK1b and UPK2 genes. RNA from human urothelial cells (HUCs) was also tested. ^**^P < 0.01 and ^***^P < 0.001 vs. day 0. ^###^P < 0.001 vs. HUCs. (**b**) Confocal microscopic images are shown. (**c**,**d**) On days 0 and 21, cells were immunostained with the indicated antibodies, while cell nuclei were also stained with Hoechst 33342. Fluorescence microscopic images (**c**) and the proportions of positive cells (**d**) are shown. ^*^P < 0.05 and ^**^P < 0.01 vs. day 0. Values are mean +/− SD (n = 3).

The cells prepared in this fashion (aHDFs transduced with FTLK and cultured in CnT-Prime medium in laminin-coated plates) were referred to as the directly converted urothelial cells (dUCs), and subjected to detailed characterization. Chronological analysis indicated that FTLK-transduced cells significantly expressed UPK1b 8 days after the gene transfer, and the expression level reached a peak on day 15 (Fig. 2a). UPK2 mRNA was elevated in FTLK-transduced cells from day 11, and peaked on day 21 (Fig. 2a). Epithelial colonies appeared on day 8, and increased in size thereafter (Fig. 2b).

We also checked expression of CDH1, a typical transmembrane protein expressed in epithelial cells, and KRT8/18, an epithelial cytoskeleton expressed in whole normal urothelium, in addition to UPK1b and UPK2 proteins^23^. Immunocytochemical analysis confirmed that approximately 42% of the cells expressed UPK1b, while UPK2-, CDH1- and KRT8/18-positive cells accounted for approximately 23%, 26% and 26%, respectively (Fig. 2c,d). The results suggested that the rate of conversion was approximately 25%.

Osborn *et al*. reported a procedure to differentiate iPS cells into urothelial cells via definitive endoderm^6^. We compared the dUCs with the iPS cell-derived induced urothelial cells (iUCs) that we prepared based on the report by Osborn *et al*. with slight modifications (Supplementary Fig. 6A). The dUCs but not iUCs strongly expressed mRNA for UPK1b and UPK2 genes, whereas iUCs expressed uroplakin 1a (UPK1a) and uroplakin 3a (UPK3a) mRNA at high levels (Supplementary Fig. 6B), strongly suggesting that dUCs resembled immature urothelial cells more closely than iUCs did.

### Pluripotent and some non-urothelial cell markers were not expressed in dUCs

To exclude the possibility that fibroblasts were temporarily converted into iPS cells or non-urothelial cells that subsequently gave rise to urothelium-like cells, we examined expression of pluripotent markers and non-urothelial cell markers in FTLK-transduced cells during the conversion from aHDFs and dUCs. As results, no significant expression of LIN28A, POU5F1, SOX2 and NANOG mRNA was detected in the FTLK-transduced cells throughout the culture periods, while NANOG protein was not detected either (Supplementary Fig. 7A,B). Similarly, the FTLK-transduced cells did not express mRNA for endoderm-derived intestinal epithelial cell marker (CDX2)^24^ and hepatocyte marker (ALB)^8^ at any time points (Supplementary Fig. 7C). Besides, the FTLK-transduced cells expressed neither a mesoderm-derived endothelial cell marker (CD31)^25^ nor an ectoderm-derived salivary gland marker (AQP5)^26^ (Supplementary Fig. 7C).

Therefore, FTLK-transduced cells did not pass through the pluripotent state on the conversion process into dUCs, and they did not convert into non-urothelial cell lineages.

### dUCs were selectively propagated through serial subcultures

The dUC colonies that appeared by day 21 after the gene transduction did not vigorously grow thereafter even if they were continuously cultured in the same plates. To obtain a larger number of dUCs of a higher purity, we harvested the cells from the initial culture (Passage 0 (P0)) and reseeded them in fresh plates for subsequent culture (P1). However, few epithelial colonies were formed and non-epithelial cells slightly proliferated, if P0 dUCs generated in the CnT-Prime were subcultured in the same medium (Supplementary Fig. 8). The “Uromedium” also failed to support generation of cobblestone-like colonies in the secondary culture (Supplementary Fig. 8). Therefore, we tried to determine the composition of the suitable culture medium for dUCs by modifying concentrations of the contents of the “Uromedium”. As shown in Supplementary Fig. 9A, the concentrations of insulin and hydrocortisone were not critical, while 1 to 10 mM IBMX played a beneficial role, in inducing FLTK-transduced cells to express UPK2 mRNA. Both hydrocortisone-free medium and medium containing 10 mM IBMX allowed FLTK-transduced cells to form epithelial colonies (Supplementary Fig. 9B). An addition of EGF at 0.01 to 1 ng/mL increased UPK2 mRNA expression (Supplementary Fig. 9A), although EGF was reportedly not required for expansion and differentiation of urothelial cells^23^. Supplementation of tranylcypromine, an irreversible monoamine oxidase inhibitor known to increase the efficiency of reprogramming from fibroblasts into iPS cells^27^, also enhanced epithelial colony formation. (Supplementary Fig. 9C).

**Figure 3:**
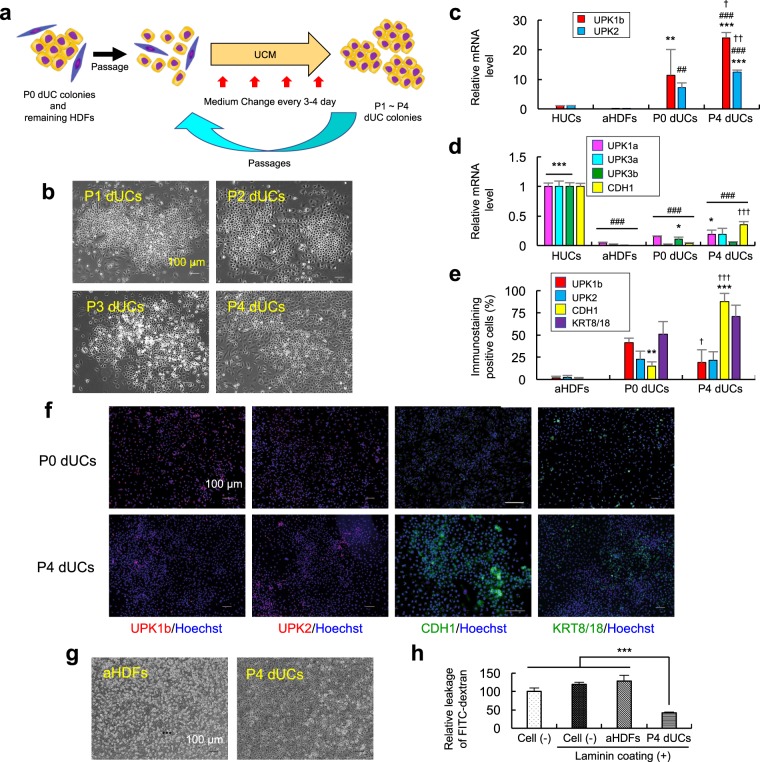
dUCs were expanded and enriched through serial passages in UCM. (**a**) Scheme of passage procedures of dUCs. Passage 0 (P0) dUCs were induced as in Fig. 1a, except that UCM was substituted for CnT-Prime. They were detached, resuspended in UCM and reseeded in fresh laminin-coated plates to obtain passage 1 (P1) dUCs. Passages were repeated to obtain P2 to P4 dUCs. (**b**) Confocal microscopic images of the P1 to P4 dUCs are shown. (**c,d**) RNA was extracted from the cells and mRNA for the indicated genes was evaluated by real-time RT-PCR. ^*^P < 0.05, ^**^P < 0.01 and ^***^P < 0.001 vs. aHDFs. ^##^P < 0.01 and ^###^P < 0.001 vs. HUCs. ^†^P < 0.05, ^††^P < 0.01 and ^†††^P < 0.001 vs. P0 dUCs. (**e,f**) Cells were immunostained with the indicated antibodies. % positive cells (**e**) and fluorescent microscopic images (**f**) are shown. ^**^P < 0.01 and ^***^P < 0.001 vs. aHDFs. ^†^P < 0.05 and ^†††^P < 0.001 vs. P0 dUCs. (**g,h**) P4 dUCs and aHDFs were cultured on inner chambers in Transwells, and permeability assay was performed using FITC-Dextran. Confocal microscopic images of the inner chamber (**g**) and percentage of leaked FITC-dextran (**h**) are shown. Values are mean +/− SD (n = 3). ^***^P < 0.001 between groups.

Based on these results, we determined that the most suitable composition of culture medium for subcultures of dUCs was as follows: EpiLife medium with 60 µM calcium supplemented with 60 μg/mL bovine pituitary extract, 5 μg/mL human recombinant insulin, 30 μg/mL gentamycin, 15 ng/mL amphotericin, 2% FBS, 0.01 ng/mL human recombinant EGF, 1 mM IBMX and 1 μM tranylcypromine. We referred to this medium as Urothelial Cell Conversion and Maintenance Medium (UCM), and found that UCM supported formation of epithelial colonies by the FLTK-transduced cells, while growth of fibroblast-like cells was suppressed (Supplementary Figs 8, 9D). Then we examined whether repetitive passages and selective growth of dUCs could be supported by this novel medium (Fig. 3a). As shown in Fig. 3b, we succeeded in expanding dUCs through at least four serial passages. Passage 4 (P4) dUCs strongly expressed UPK1b, UPK2 and CDH1 but not UPK1a, UPK3a and uroplakin 3b (UPK3b) mRNA (Fig. 3c,d), suggesting that dUCs do not mature through the passage in UCM. Immunocytochemical analysis showed that UPK1b-, UPK2-, CDH1- and KRT8/18-positive cells accounted for approximately 19%, 21%, 88% and 71% of P4 dUCs, respectively (Fig. 3e,f). These results suggest that dUCs were more effectively expanded than fibroblast-like cells, and stably remained urothelium-like, during passages in our UCM.

### Passage 4 dUCs exerted significant barrier functions *in vitro*

Next, we checked the barrier function of P4 dUCs by *in vitro* permeability assay using Transwells. P4 dUCs were allowed to form a confluent sheet on laminin-coated bottom membrane of the inner chamber (Fig. 3g), and leakage of FITC-dextran from the inner to outer chamber was evaluated. Compared with cell-free control and another control in which aHDFs were cultured, the rate of permeability of FITC-dextran was significantly smaller in the Transwells with dUCs (Fig. 3h). HUCs and terminally differentiated HUCs also decelerated permeabilization of FITC-dextran (Supplementary Fig. 10). Therefore, it was suggested that P4 dUCs prepared by our procedure formed epithelial barrier *in vitro*.

### RNA-seq further confirmed urothelial features of the P2 dUCs

**Figure 4:**
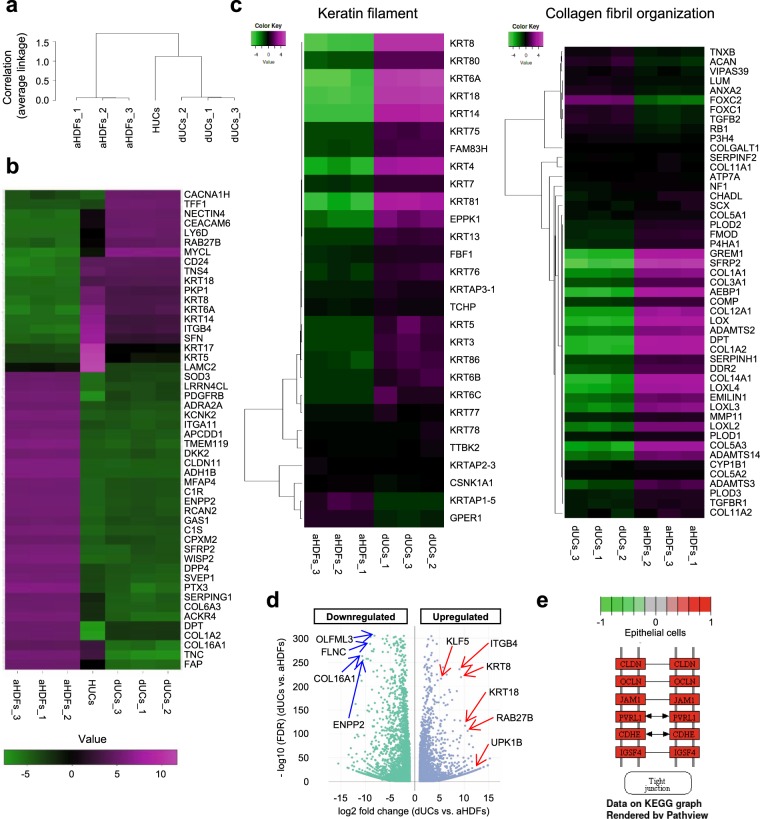
Transcriptome analysis of passage 2 (P2) dUCs. Total RNA was extracted from P2 dUCs (n = 3), aHDFs (n = 3) and HUCs (n = 1) and subjected to RNA-seq analysis using Illumina Novaseq. 6000 (Illumina). (**a**) Hierarchical clustering sample tree analysis showing top 75% genes with maximum expression levels. (**b**) Heatmap of hierarchical clustering analysis showing the most variable top 50 genes. (**c**) Heatmaps for GO genesets comparing P2 dUCs with aHDFs are shown. (**d**) Volcano plot of differential gene expression analysis showing comparison between dUCs and aHDFs. Some genes specific to urothelial cells and fibroblasts were indicated by the red and blue arrows, respectively. (**e**) The KEGG pathway diagram comparing dUCs and aHDFs. Red color shows the genes highly expressed in dUCs, while green color shows genes highly expressed in aHDFs.

Similarity between Passage 2 (P2) dUCs and urothelial cells was analyzed by RNA sequencing (RNA-seq). Hierarchical clustering analysis of genome-wide gene expression profiles revealed that dUCs were more similar to HUCs than to aHDFs (Fig. 4a). Figure 4b shows data of the most variable fifty genes, demonstrating that both dUCs and HUCs expressed urothelium-related genes including urothelium-related keratins (KRT8, 18, 5, 17, 6 A and 14) and RAB27B that is involved in transportation of uroplakins towards apical membrane^28^. In contrast, fibroblast-related genes (FAP, COL16A1, COL1A2, DPT, PTX3, etc.) were down-regulated in dUCs and HUCs (Fig. 4b). Differential gene expression analysis further confirmed that dUCs strongly expressed urothelium-related genes but not fibroblast-related genes (Fig. 4c,d). HUCs and dUCs expressed similar expression patterns of genes that are related to keratin filament and collagen fibril organization (Supplementary Fig. 11). Notably, dUCs expressed KLF5 that is one of the important factors for the development of bladder urothelium (Fig. 4d)^29^. dUCs also expressed epithelial tight-junction-related genes at high levels as shown by KEGG pathway analysis (Fig. 4e).

### dUCs were recruited to regenerating urothelium lining *in vivo*

**Figure 5:**
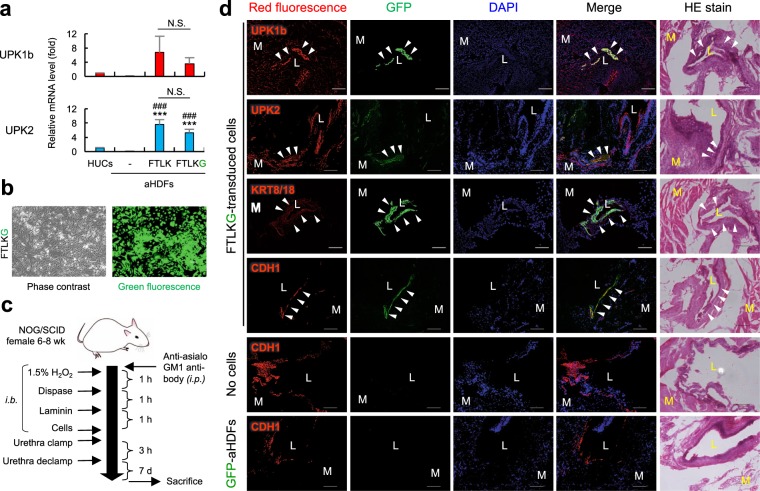
Transplantation of FTLKG-transduced cells to urothelium-injured mouse bladder resulted in localization of GFP-labeled dUCs at the urothelial mucosa. (**a**,**b**) aHDFs were transduced with FTLK and GFP retroviral vectors (FTLKG) and cultured in CnT-Prime for 21 days as in Fig. 3. mRNA levels for the indicated genes were measured by real-time RT-PCR (**a**), while representative phase contrast and fluorescent microscopic images are shown (**b**). ^***^P < 0.001 vs. non-transduced aHDFs. ^###^P < 0.001 vs. HUCs. N.S.: not significant between groups. (**c**,**d**) FTLKG-transduced aHDFs were cultured for 4 days in Standard Medium and transplanted into urothelium-injured bladder of NOG/SCID mice through trans-urethral catheter (**c**). One week after the transplantation, mice were sacrificed. Urinary bladder tissue specimens were subjected to immunohistochemical analysis using the indicated antibodies and nuclear staining with DAPI, while serial sections were stained with HE (**d**) (n = 4 mice per group). i.b., intra-bladder; i.p., intra-peritoneal. M and L represent muscle and lumen of the bladder, respectively. Arrowheads represent GFP-labeled dUCs.

We examined whether FTLK-transduced aHDFs can be successfully converted into dUCs *in vivo* and contribute to regeneration of urothelium. aHDFs transduced with FTLK and GFP genes (FTLKG) were converted into GFP-labelled dUCs if they were cultured in CnT-Prime for 21 days (Fig. 5a,b). For the *in vivo* experiments, the aHDFs cells were transplanted, 4 days after the FTLKG gene transduction, into a damaged bladder of the mice with interstitial cystitis^30^ (Fig. 5c,d). One week after the transplantation, GFP-labeled cells were demonstrated at the inner surface of bladder, and they expressed UPK1b, UPK2, CDH1, and KRT8/18 (Fig. 5d) as well as KRT20 that is generally expressed in terminal differentiated umbrella cells (Supplementary Fig. 12). In contrast, intra-bladder transplantation of GFP-labeled aHDFs did not result in generation of GFP-positive CDH1-expressing cells (Fig. 5d). Therefore, FLTKG-transduced aHDFs were converted into dUCs *in vivo* in injured bladder urothelium and participated in the regeneration of the tissue.

Finally, we examined potential tumorigenicity of dUCs. So far as we tested, FLTK-transduced aHDFs did not form a tumor after they were subcutaneously inoculated into immunodeficient mice (Supplementary Fig. 13A,B).

## Discussion

Direct conversion may efficiently convert somatic cells that can be obtained from patients with minimal invasion into functional tissue cells that could be safely transplanted back into the patients. In our study, urothelial cells were directly converted from human dermal fibroblasts by transducing four defined transcriptional factors, FOXA1, TP63, MYCL and KLF4, followed by culture under appropriate conditions.

The FOXA1 and TP63 are known to play important roles in urothelial development. FOXA1 is expressed during mouse urothelium development^31^ and acts as a pioneer factor that binds to chromatins to modulate their structure and regulate gene expression^32^. At the promotor regions of uroplakin genes, specific binding sites for FOXA1 are present^17^. TP63 is a well-known basal epithelial marker and required for development of epithelium including urothelium^18^. Urothelial basal cells that express TP63 are considered as stem cells of the urothelial tissue^33^.

It may be reasonable to suppose that KLF4 and MYCL support mesenchymal-to-epithelial transition^34^. As a member of the Myc family genes, MYCL has been shown to play crucial roles in the generation of iPS cells. MYCL is reportedly far less tumorigenic than MYC^35^. KLF4 is also one of the main transcriptional factors that are used for inducing iPS cells^36^. KLF4 activates expression of some epithelial markers including CDH1, and facilitates mesenchymal-epithelial transition^34^. KLF4 is significantly expressed in the bladder urothelium during mouse development^37^ and acts as a tumor suppressor^38^.

We examined UPK1b and UPK2 as urothelial cell markers. Though UPK1b is expressed in whole urothelium, it is also expressed in the cornea and conjunctiva^39^. In contrast, UPK2 is one of the most specific urothelial cell markers^22^. UPK2 is strongly expressed in terminally differentiated umbrella cells, but is also expressed weakly in intermediate and basal cells^22,40,41^. We consider that dUCs remain at an immature stage of urothelial cells, judging from their high proliferative ability and low expression of other terminal differentiation markers, UPK1a, UPK3a and UPK3b. This might be partially due to the expression of transgenic TP63 that is an important transcriptional factor for urothelial basal cells. If we use plasmid vectors instead of retroviral vectors, TP63 may be silenced in the dUCs, so that more mature dUCs could be induced.

To our knowledge, this study is the first to successfully transplant non-cancerous urothelial cells into bladder lumen. FTLK-transduced aHDFs were converted into urothelial cells under *in vivo* environment, because the cells were cultured in Standard Medium for only 4 days before transplantation. This may imply the possibility that our procedure could be applicable to treatment of bladder and urinary tract disorders such as interstitial cystitis, neurogenic bladder, congenital anomalies including vesicoureteral reflux, vesico-vaginal fistula, and bladder injury.

As a source of the somatic cells to be directly converted, we used dermal fibroblasts derived from a human adult. Dermal fibroblasts have a high proliferation capacity *in vitro* regardless of the age of the donors^42^, so that our procedures may be applicable to elderly people with a high incidence of bladder disorders.

## Materials and Methods

### Culture media

High-glucose DMEM supplemented with 10% FBS (Gibco), MEM non-essential amino acids, 100 mM sodium pyruvate and 100 U/mL penicillin-streptomycin was used as “Standard Medium”. CnT-Prime was purchased from CELLnTEC. “Uromedium” was prepared according to Osborn *et al*.^6^ with slight modifications. Briefly, “Uromedium” was comprised of EpiLife medium with 60 µM calcium (Gibco; MEPI500CA) supplemented with 60 μg/mL bovine pituitary extract (Gibco), 5 μg/mL human recombinant insulin (Diagnocine), 500 ng/mL hydrocortisone (Tokyo Chemical Industry), gentamicin/amphotericin (Gibco), 2% FBS, 0.1 ng/mL human recombinant epidermal growth factor (EGF) (RSD) and 100 μM 3-Isobutyl 1-methylxanthine (IBMX) (Sigma-Aldrich). “UCM” was comprised of EpiLife medium with 60 µM calcium supplemented with 60 μg/mL bovine pituitary extract, 5 μg/mL human recombinant insulin, gentamycin/amphotericin, 2% FBS, 0.01 ng/mL human recombinant EGF, 1 mM IBMX and 1 μM tranylcypromine (Abcam).

### Cells

aHDFs and Plat-GP cells were purchased from ScienCell Research Laboratories and Cell Biolabs, respectively, and cultured in Standard Medium. aHDFs at passage number less than 10 were used for experiments. HUCs derived from a 24-years-old male African American were purchased from KURABO Industries (KP-4309; Lot 04101). Induced pluripotent stem (iPS) cells were purchased from JCRB Cell Bank and maintained as previously described^43^. Briefly, iPS cells were cultured in StemFit medium (Ajinomoto; AK02N) on Easy iMatrix-511 silk (Nippi; 0.25 μg/cm^2^; preincubated for 1 h at 37 °C)-coated plates with. For passage, iPS cells were dissociated into a single cell suspension in StemFit medium with Rock inhibitor (Nacalai Tesque; Y-27632) and reseeded in new culture plates. On the next day, medium was replaced by fresh StemFit medium without Rock inhibitor, followed by medium change every other day. Passage was performed when iPS cells reached 80% to 90% confluency. T24, human bladder carcinoma cell line, was a kind gift from professor Ashihara (Kyoto Pharmaceutical University, Kyoto, Japan), and cultured in RPMI1640 medium (Nacalai Tesque) supplemented with 10% FBS, MEM nonessential amino acids, 100 mM sodium pyruvate and 100 U/mL penicillin-streptomycin.

### Antibodies

As primary antibodies for immunostaining, rabbit polyclonal anti-uroplakin 1b IgG (Abcam; #ab102961; dilution 1:100), rabbit polyclonal anti-uroplakin 2 IgG (Proteintech; #21149-1-AP; 1:100), mouse monoclonal anti-E-cadherin IgG (BD Biosciences; #610181; 1:50), rabbit polyclonal anti-Nanog IgG (Reprocell; #09-0020; 1:200), mouse monoclonal anti-keratin 8/18 IgG (CST Japan; #4546 S; 1:50) and rabbit monoclonal anti-Keratin 20 (KRT20) IgG (CST Japan; #13063; 1:100) antibodies were used. As secondary antibodies, the following antibodies were used. Alexa Fluor 488-conjugated goat anti-rabbit IgG and anti-mouse IgG antibodies (Life technologies; #A11034 and #11029, respectively; 1:500), and Alexa Fluor 594-conjugated goat anti-rabbit IgG and anti-mouse IgG antibodies (Life technologies; #A11037 and #11032, respectively; 1:500).

### Retroviral vectors

Full length cDNA clones for human FOXA1, IRF1, TP63 and SHH genes were purchased from DNAFORM library, and coding sequences were amplified by RT-PCR with KOD-Plus-Neo DNA polymerase (Toyobo) and primers specific for FOXA1 (5′-CAGCAGTGTGGTGGTACGGGATGTTAGGAACTGTGAAGATG-3′ and 5′-ACCGGCGCTCAGCTGGCTAGGAAGTGTTTAGGACGGG-3′), IRF1 (5′-CAGTGTGGTGGTACGGGATGCCCATCACTCGGATGCGC-3′ and 5′-ACCGGCGCTCAGCTGGACCGGCGCTCAGCTGG-3′), TP63 (5′-CAGTGTGGTGGTACGGGATGAATTTTGAAACTTCACGG-3′ and 5′-ACCGGCGCTCAGCTGGCTATCACTCCCCCTCCTCTTT-3′) and SHH (5′-CAGTGTGGTGGTACGGGATGCTGCTGCTGGCGAGATGT-3′ and 5′-ACCGGC GCTCAGCTGGCTAGCTGGACTTGACCGCCAT-3′). The amplified fragments were cloned into pMxs retroviral plasmid vector (Cell Biolabs) using the Gene Art System (Invitrogen). pMxs plasmids containing human POU5F1, KLF4, MYCL and GFP genes were kind gifts from professor Yamanaka (CiRA, Kyoto University, Kyoto, Japan). Plat-GP cells were seeded in 100 mm plates at a density of 3 × 10^6^ cells/dish and cultured for 24 hours. Cells were then transfected with 5.0 μg of a pMxs-based vector mentioned above and 2.5 μg of pCMV-VSV-G using X-tremeGENE 9 DNA Transfection Reagent (Sigma-Aldrich). After 24 hours the medium was replaced by antibiotic-free Standard Medium. The supernatant of the culture was harvested and used as a retroviral vector suspension.

### Conversion of fibroblasts into dUCs

aHDFs were seeded into 12-well plates that had been coated with either atelocollagen (KOKEN; I-PC 30), poly-L-lysine (ScienCell) or laminin (Easy iMatrix-511 silk) at a density of 3 × 10^4^ cells/well. Twenty-four hours later, the medium was replaced by the retroviral vector suspension that had been filtrated through a 0.45 μm-pore filter (Toyo Rochi Kaisha, Ltd., ADVANTEC) and supplemented with polybrene at 4 μg/mL. On the next day culture supernatant was replaced by virus-free Standard Medium, followed 3 days later by replacement by CnT-Prime or “UCM”. Culture medium was changed every 3 or 4 days.

### Passages of dUCs

dUCs were passaged when dUCs reached 60 to 80% confluency or 14 days after the previous passage. dUCs were detached using trypsin/EDTA (Nacalai Tesque) and Cell Scraper (IWAKI), reseeded in laminin-coated 12-well plates at a density of 1 × 10^4^ cells/well, and cultured in “UCM”.

### Induction of urothelial cells from iPS cells

iPS cells were converted into urothelial cells as previously described with slight modifications^6^. Briefly, iPS cells were seeded in laminin-coated 6-well plates at a density of 2 × 10^4^ cells/well and cultured until they reached 60 to 70% confluency. Medium was replaced by RPMI1640 supplemented with 2% FBS, GlutaMAX (Gibco), 100 U/mL penicillin-streptomycin and 5 μM IDE1 (Cayman). Medium was replaced by fresh one every 1–2 days. Nine days after induction of definitive endoderm (DE) cells, medium was replaced by “Uromedium” to allow differentiation of DE cells into urothelial cells. Medium was changed every 2–3 days for 18 days, followed by culture with 10 mM Rosiglitazone (Sigma-Aldrich) and without EGF for 3 days.

### Real-time RT-PCR

Total RNA was extracted from cells using RNeasy Mini Kit (Qiagen) according to the manufacturer’s instruction. Total RNA of early passage HUCs was purchased from ScienCell Research Laboratories (#4325, Lot 4740). Total RNA of human small intestine from five Caucasians aged 20 to 61 (#636539, Lot 1611206 A) and of human liver from four Asian and Caucasian aged 26 to 78 (#636531, Lot 1703002) was purchased from Clontech Laboratories. Total RNA of human salivary glands from 87-years-old female was purchased from BioChain (R1234212-10; Lot B111155), while total RNA of human umbilical vein endothelial cells was purchased from PromoCell (C-12200). cDNA was synthesized using ReverTra Ace qPCR (TOYOBO Life Science), and subjected to real-time RT-PCR using StepOnePlus Real-Time PCR Systems (Applied Biosystems). Reaction mixture included Taqman probe (Applied Biosystems) and Taqman Fast Advanced Master Mix (Applied Biosystems). The relative mRNA levels were calculated as follows: Relative mRNA level (fold) = [(target gene mRNA level in sample)/(β-actin gene mRNA level in sample)]/[(target gene mRNA level in control)/(β-actin gene mRNA level in control)].

### Immunocytochemical analysis

Cells cultured in 12 well plates were washed by PBS(-) and fixed in 4% paraformaldehyde (PFA) for 30 minutes, followed by permeabilization using 0.2% Triton-X 100 (Nacalai Tesque) for 15 minutes at room temperature. Background staining was blocked by Blocking One Histo (Nacalai Tesque) for 30 minutes at room temperature, followed by incubation with a primary antibody overnight at 4 °C. After washing, secondary antibodies were added and cells were incubated for 1 h at room temperature in the dark. Cells were washed and cell nuclei were stained with Hoechst 33342 (Dojindo) for 5 minutes. Cells were observed by BZ-X (Keyence). Each immunostaining-positive and -negative cells were counted by BZ-X Analyzer software (Keyence). The percentage of immunostaining-positive cells were calculated as follows: % immunostaining cells = (the number of immunostaining (+) Hoechst 33342 (+) cells)/(total number of Hoechst 33342 (+) cells) × 100.

### RNA sequencing

Total RNA was extracted from aHDFs and P2 dUCs (cultured in UCM) with the RNeasy Mini Kit (QIAGEN) according to the manufacturer’s instruction. Total RNA from human urothelial cells (HUCs) was purchased from ScienCell Research Laboratories. Quality of each RNA sample was check by agarose gel electrophoresis (gel concentration: 1%, voltage: 180 V, run time: 16 min) and Agilent 2100 analysis. Library preparation and sequencing were performed by Novogen Co., Ltd. Briefly, ribosome RNA was removed using the Ribo-Zero kit. The mRNA was randomly fragmented by an addition of fragmentation buffer, followed by cDNA synthesis. The qualified libraries were sequenced with Illumina Novaseq 6000 (Illumina; paired-end, 150 bp). Filtered reads were mapped to reference genome (GRCh38) using HISAT2 version 2.1.0 (https://ccb.jhu.edu/software/hisat2/index.shtml). After removing residual ribosome RNA from read assignments with bedtools (https://bedtools.readthedocs.io/en/latest/), mapped reads were counted using featurecounts (http://bioinf.wehi.edu.au/featureCounts/)^44^ with the annotation GTF file from GENCODE (GENCODE 29; https://www.gencodegenes.org/). Analysis was performed using iDEP8.1^45^. Read count data were normalized by counts per million function in edgeR, and the genes that did not have more than 0.5 counts per million in at least one sample were removed. Filtered count data were then performed started log transformation in edgeR. Transformed data was used for exploratory analyses (hierarchical clustering analysis and differential gene expression analysis), and non-transformed data were used for pathway analysis that was performed using GSEA (Gene Set Enrichment Analysis) method with fold-change values returned by DESeq2. Gene expression data was visualized on KEGG pathway diagrams^46^ using Pathview^47^.

### *In vitro* permeability assay

HTS Transwell clear 12-well plates were purchased from Corning, and the inner chambers, which contained polycarbonate membrane 12 mm in diameter with 0.4 μm pores, were coated with Easy iMatrix-511 silk (Nippi; 0.25 μg/cm^2^; preincubated for 1 h at 37 °C). P4 dUCs or aHDFs were resuspended in UCM and seeded in the inner chamber at a density of 1 × 10^5^ cells/well. HUCs were resuspended in Urolife D Complete Medium (Urolife) and seeded in the inner chamber at a density of 1 × 10^5^ cells/well. For terminal differentiation, some aliquots of HUCs were cultured in Urolife D Complete Medium supplemented with 1 μM Rosiglitazone and 1 μM PD153035 for 4 days^48^. Cells were cultured for 5 days followed by washing with PBS(-). FITC-Dextran with an average molecular weight of 4 kDa (IWAI Chemicals) was added to the inner chamber at a concentration of 2 mg/mL. After incubation for 20 min in the dark, the supernatant in the outer chamber was transferred into 96-well plates, and fluorescence intensity in each well was measured by SpectraMax M2 (Molecular Devices) with excitation and emission wave lengths at 535 nm and 485 nm, respectively. Percentage of leaked FITC-dextran was calculated as follows: (Fluorescent count of each well)/(Fluorescent count of no cell control well) × 100.

### Transplantation of dUCs into mouse bladder

All animal experiments were approved by the Committee for Animal Research, Kyoto Prefectural University of Medicine (M30–281). The care of the animals was in accordance with the institutional guideline and Guide for the Care and Use of Laboratory Animals. Female NOG/BALB-Rag2^null^IL-2Rγ^null^/NSG (NOG/SCID) mice were purchased from CLEA Japan. An interstitial cystitis model of mice^30^ and intra-bladder cell transplantation procedure^49^ were previously described with slight modifications (Fig. 5c). The mice at the age of 6 to 8-weeks were anesthetized and intraperitoneally injected with anti-asialo GM1 antibody at a dose of 100 mg/mouse. At the same time, a 24-gauge catheter was inserted into the bladder through the urethra, and 50 μL of 1.5% hydrogen peroxide solution was infused into the bladder. One hour later, the bladder was rinsed with PBS(-), and 50 μL solution containing 12,000 PU dispase II (FUJIFILM Wako Pure Chemical Corporation) was infused into the bladder, followed 1 h later by an administration of Easy iMatrix-511 silk for 1 h. aHDFs that had been transduced with FTLK and cultured for 4 days in Standard Medium were resuspended in PBS(-) at a density of 3 × 10^6^ cells/100 μL and transplanted into the bladder (n = 4 each). Other groups of animals were given non-transduced aHDFs (3 × 10^6^ cells/100 μL of PBS(-)) or cell-free PBS (-) into the bladder. The mouse urethra was ligated, and 3 h later ligated suture was released to allow spontaneous voiding.

### Assessment of tumorigenicity *in vivo*

Six- to eight-week-old female NOG/SCID mice were anesthetized and given an intraperitoneal injection with 100 mg of anti-asialo GM1 antibody. aHDFs that had been transduced with FTLK and cultured for 4 days in Standard Medium were mixed with Matrigel (Corning) and percutaneously injected into the mice at a density of 1 × 10^7^ cells/100 μL. T24 cells and non-transduced aHDFs were used as a positive and negative control, respectively. Volumes of tissue swelling at the injected sites or volumes of tumors if generated, were measured with a micrometer caliper every week for 10 weeks.

### Immunohistochemistry

Urinary bladder was removed from the mice 7 days after the transplantation. After fixation in formaldehyde, some parts of the organ were cryoprotected in 30% sucrose PBS (-) and freeze-mounted. Other parts were embedded in paraffin. Specimens were sliced into 5 or 10 μm thick sections. After blocking of endogenous peroxidase with 3% hydrogen peroxide for 5 minutes, tissue sections were incubated with Protein Block (Dako) for 30 minutes. After washing, tissue sections were incubated with primary antibodies at 4 °C overnight. Sections were then washed and incubated with Alexa Fluor 594-conjugated goat anti-rabbit IgG or anti-mouse IgG antibodies for 60 minutes at room temperature. After washing 3 times, tissue sections were mounted using VECTASHIELD mounting medium with DAPI (Vector Laboratories, CA). Immunofluorescent staining was visualized by BZ-X (Keyence).

### Statistical analysis

Each data is expressed as mean +/− SD. Statistical significance was analyzed by Student’s *t* test or one-way ANOVA with the Tukey-Kramer post hoc test. P < 0.05 was considered significant. All analyses were conducted with EZR^50^.

## Supplementary information

Supplementary Information
