# Supplementary material for: Direct conversion of fibroblasts into urothelial cells that may be recruited to regenerating mucosa of injured urinary bladder
Source: Sci Rep. 2019 Sep 25;9:13850. doi: 10.1038/s41598-019-50388-6 (PMC6761134; doi:10.1038/s41598-019-50388-6)
Supplement: Supplementary file 1 — Supplementary Information [file 41598_2019_50388_MOESM1_ESM.pdf]

Supplementary Information

Direct conversion of fibroblasts into urothelial cells that may be recruited to regenerating mucosa of injured urinary bladder.

Yuta Inoue[1, 2], Tsunao Kishida[1], Shin-ichiro Kotani[1], Mika Akiyoshi[1, 3], Hideto Taga[1, 2], Makoto Seki[1, 3], Osamu Ukimura[2] and *Osam Mazda[1]

[1]Department of Immunology, Kyoto Prefecture University of Medicine, Kamigyo-ku, Kyoto 602-8566, Japan

[2]Department of Urology, Kyoto Prefecture University of Medicine, Kamigyo-ku, Kyoto 602-8566, Japan

[3]CellAxia Inc. 1-10-9-6F Nihonbashi Horidome-cho, Chuo-ku, Tokyo 103-0012, Japan

Corresponding Author: Osam Mazda. Department of Immunology, Kyoto Prefecture University of Medicine, Kamigyo-ku, Kyoto 602-8566, Japan. +8175-251-5329. mazda@koto.kpu-m.ac.jp

List of acronyms

iPS cell: induced pluripotent stem cell

dUC: directly converted urothelial cell

TF: transcriptional factor

aHDF: adult human dermal fibroblast

FTLK: FOXA1, TP63, MYCL, KLF4

FIT: FOXA1, IRF1, TP63

FIH: FOXA1, IRF1, SHH

FT: FOXA1, TP63

FLK: FOXA1, MYCL, KLF4

TLK: TP63, MYCL, KLF4

iUC: iPS cell-derived induced urothelial cell

UCM: Urothelial Cell Conversion and Maintenance Medium

DEGs: differentially expressed genes

FTLKG: FOXA1, TP63, MYCL, KLF4, GFP

Supplementary Figures

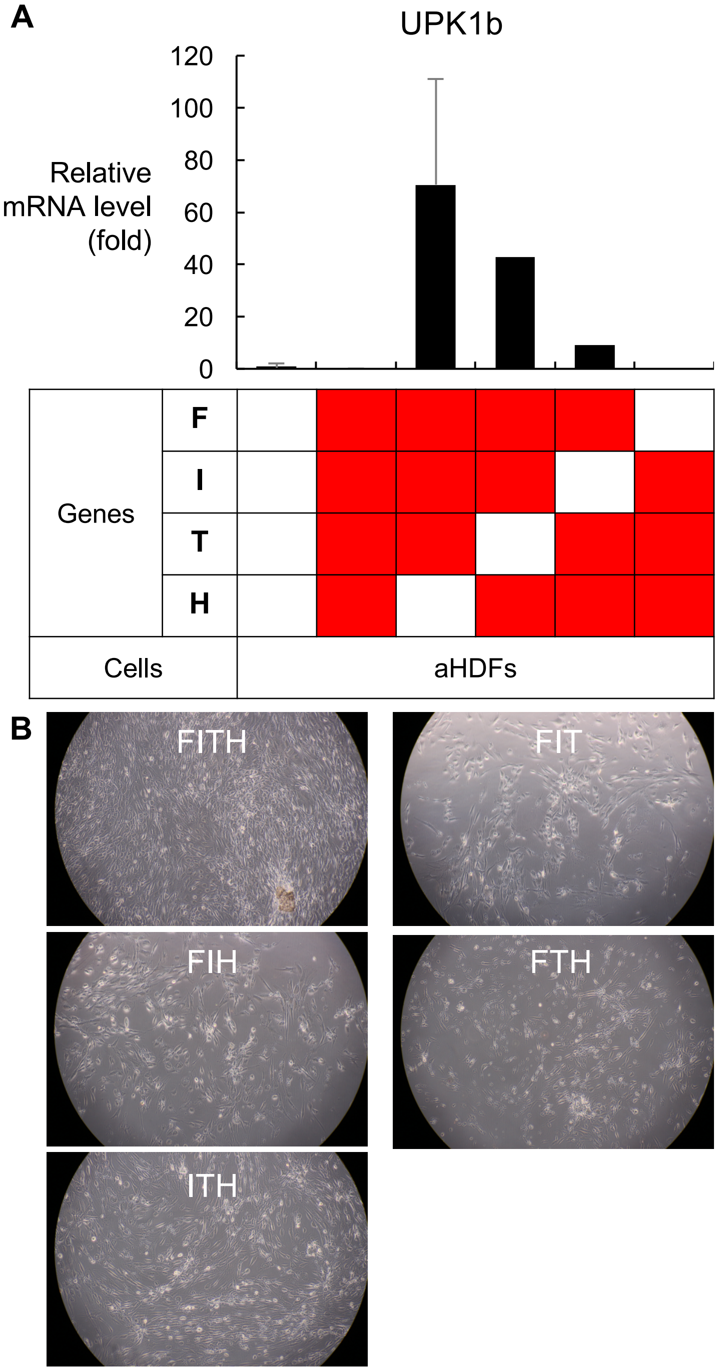

Supplementary Figure 1 | Transduction with urothelium-related TF genes was not sufficient to induce urothelium-like phenotypes in fibroblasts.

aHDFs were seeded in non-coated 12-well plates and transduced with FOXA1 (F), IRF1 (I), TP63 (T) and/or SHH (H) genes as indicated (in lower part in A, red color represents infection of each corresponding retroviral vector). Cells were cultured in Standard Medium (days 1 to 3) and CnT-Prime (days 4 to 21). (**A**) RNA was extracted from the cells and real-time RT-PCR was performed to evaluate mRNA levels for UPK1b gene. Values (averages +/- SD) were normalized to β-actin mRNA and expressed relative to values for the non-transduced control (set to 1.0) (n=3). (**B**) Representative phase contrast microscopic images are shown. Original magnification: x 10. The abbreviations used are: F, FOXA1; I, IRF1; T, TP63 and H, SHH.

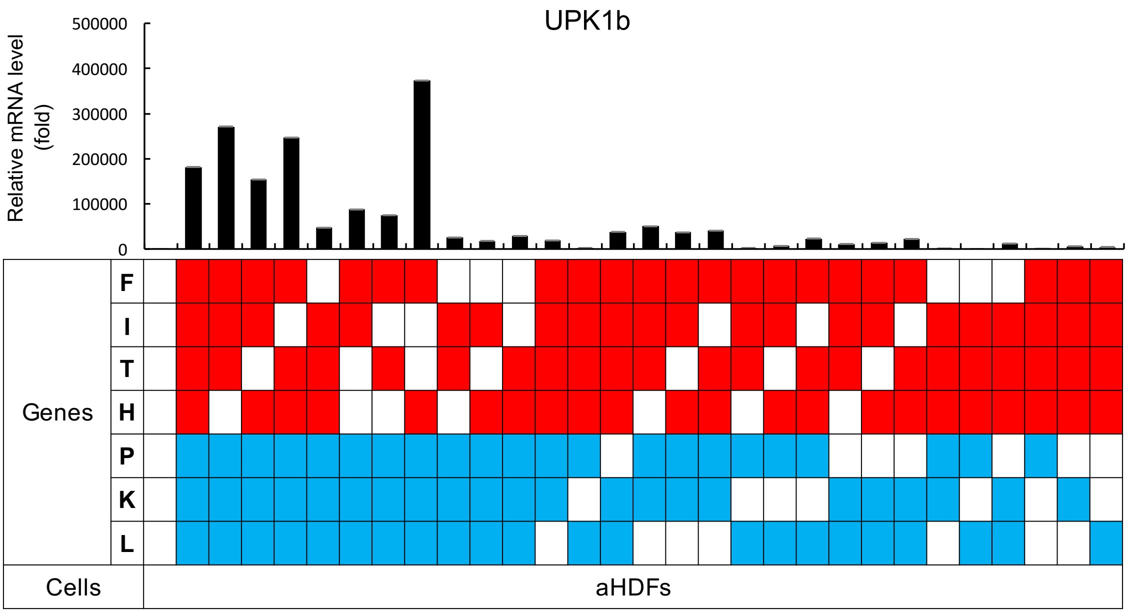

Supplementary Figure 2 | Some combinations of transcriptional factors induced UPK1b expression in aHDFs.

aHDFs were seeded in laminin-coated 12-well plates and transduced with F, I, T, H, POU5F1 (P), KLF4 (K), and/or MYCL (L) genes (in lower part, red and aqua color represents infection of each corresponding retroviral vector). Cells were cultured in Standard Medium (days 1 to 3) and CnT-Prime (days 4 to 21). On day 21, total RNA was extracted from each well, and UPK1b mRNA levels were evaluated by real-time RT-PCR. Values were normalized to β-actin mRNA and expressed relative to values for the non-transduced control (set to 1.0) (n=1). The abbreviations used are: F, FOXA1; I, IRF1; T, TP63, H, SHH; P, POU5F1; K, KLF4; and L, MYCL.

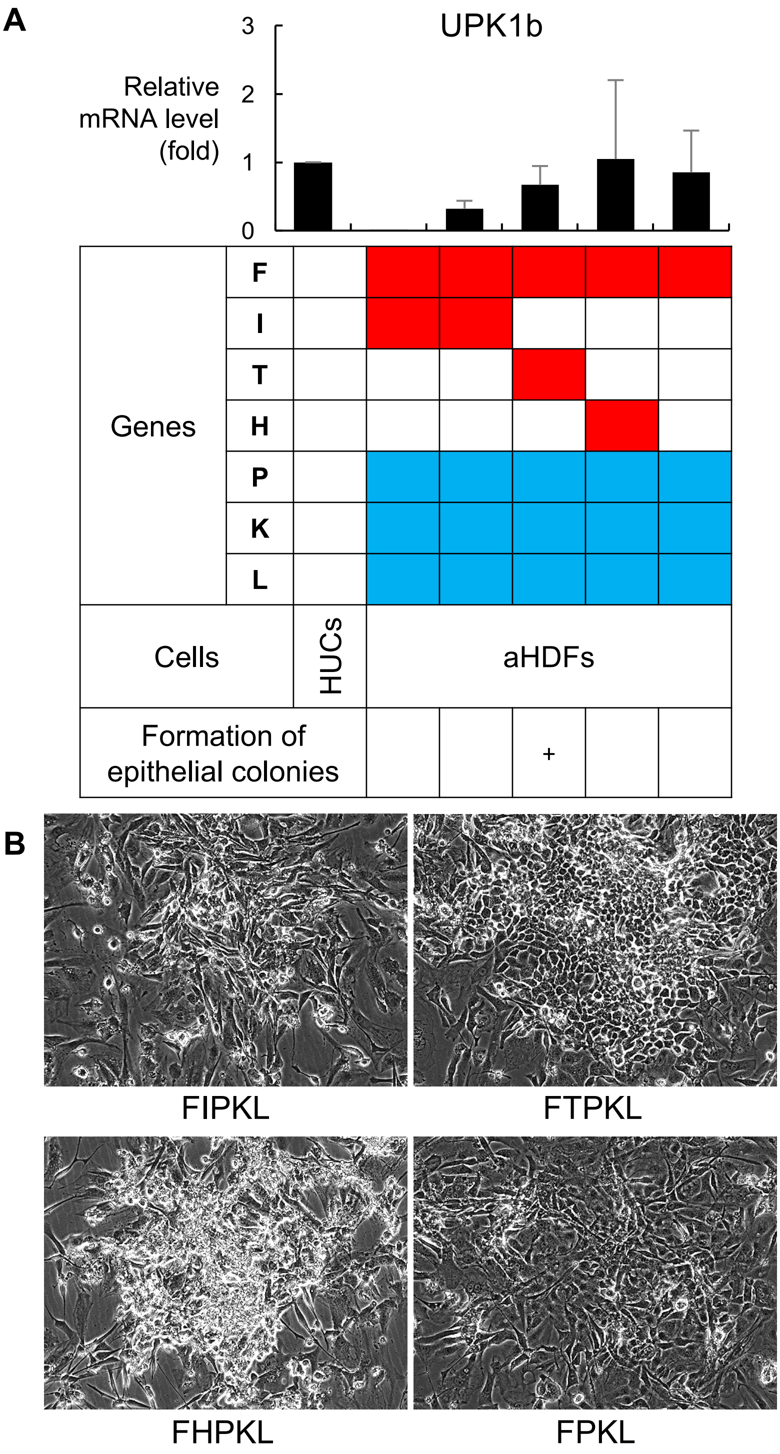

Supplementary Figure 3 | Transduction with FTPKL resulted in formation of epithelial colonies expressing UPK1b.

aHDFs were seeded in laminin-coated 12-well plates and transduced with the genes indicated in lower part of (**A**) by red or aqua color. Cells were cultured in Standard Medium (days 1 to 3) and CnT-Prime (days 4 to 21). (**A**) RNA was extracted from the cells and real-time RT-PCR was performed to evaluate mRNA levels for UPK1b gene. mRNA from HUCs was also tested. Values (averages +/- SD) were normalized to β-actin mRNA and expressed relative to values for the HUCs (set to 1.0) (n=3). (**B**) Representative phase contrast microscopic images are shown. Original magnification: x 20. The abbreviations used are: F, FOXA1; I, IRF1; T, TP63, H, SHH; P, POU5F1; K, KLF4; and L, MYCL.

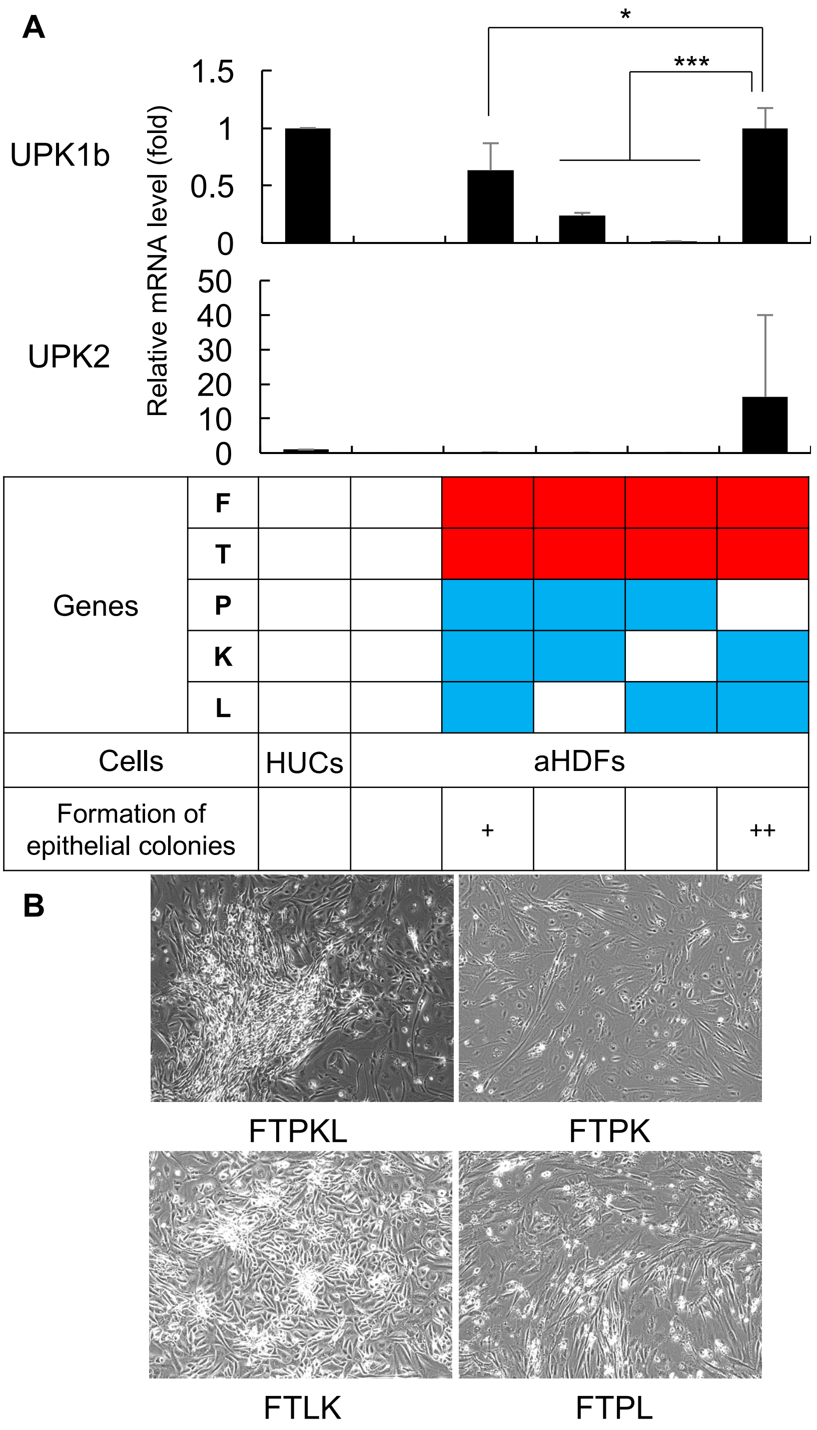

Supplementary Figure 4 | Transduction with FTLK was sufficient for induction of epithelial colonies expressing UPK1b and UPK2.

aHDFs were seeded in laminin-coated 12-well plates and transduced with the genes indicated in lower part of (**A**) by red or aqua color. Cells were cultured in Standard

Medium (days 1 to 3) and CnT-Prime (days 4 to 21). (**A**) RNA was extracted from the cells and real-time RT-PCR was performed to evaluate mRNA levels for UPK1b and UPK2 gene. mRNA from HUCs was also tested. Values (averages +/- SD) were normalized to β-actin mRNA and expressed relative to values for the HUCs (set to 1.0) (n=3). $^{*}P<0.05$ and $^{***}P<0.001$ between groups. (**B**) Representative phase contrast microscopic images are shown. Original magnification: x 10. The abbreviations used are: F, FOXA1; T, TP63, P, POU5F1; K, KLF4; and L, MYCL.

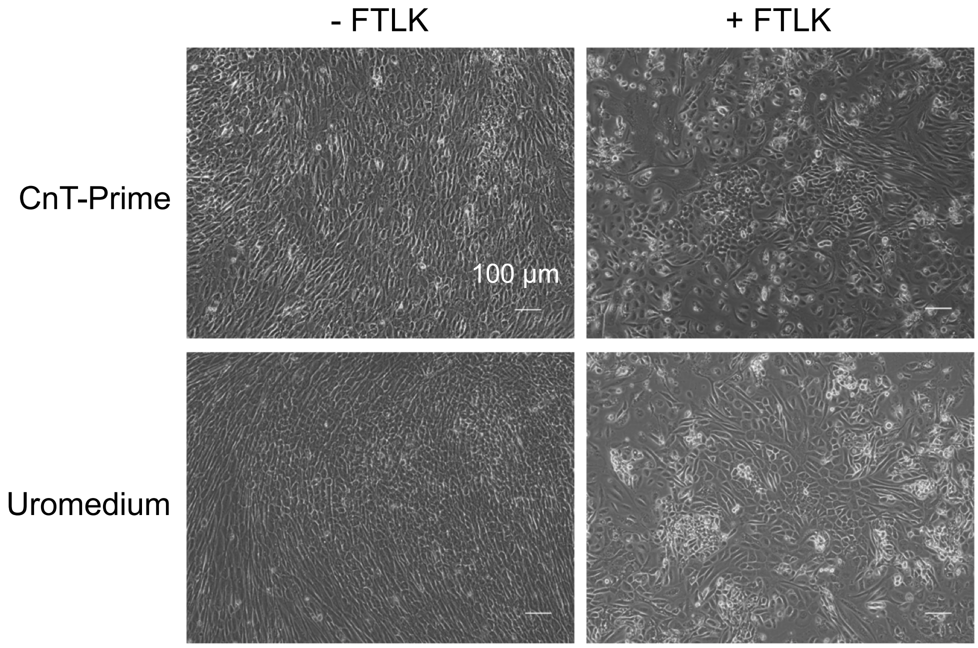

Supplementary Figure 5 | dUCs were successfully passaged depending on culture media.

FTLK-transduced aHDFs were cultured in Standard Medium (days 1 to 3) and the indicated culture media (days 4 to 21). Phase contrast microscopic images of the cells are shown.

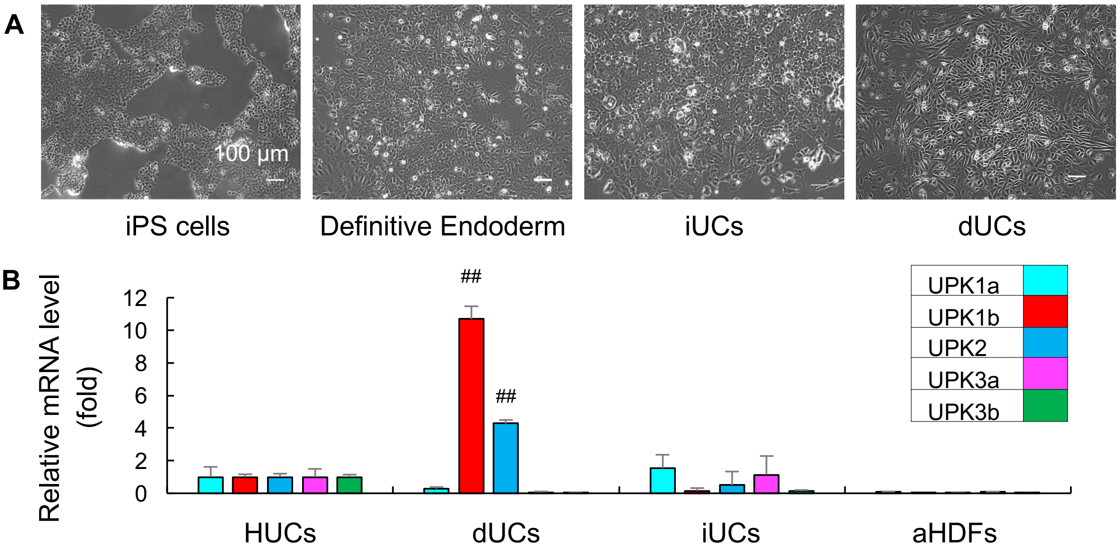

Supplementary Figure 6 | dUCs were more closely similar to immature urothelial cells than iUCs were.

iPS cells were differentiated into definitive endoderm which was subsequently differentiated into iUCs. dUCs (day 21 after gene transduction) were prepared as in Fig. 2. (**A**) Representative phase contrast microscopic images of the indicated cells are shown. (**B**) Total RNA was extracted from the indicated cells and analyzed by real-time RT-PCR. mRNA from HUCs was also tested. $^{\#\#}P<0.01$ vs. iUCs. Values (averages +/- SD) (n=3) were normalized to β-actin mRNA and expressed relative to values for HUCs (set to 1.0).

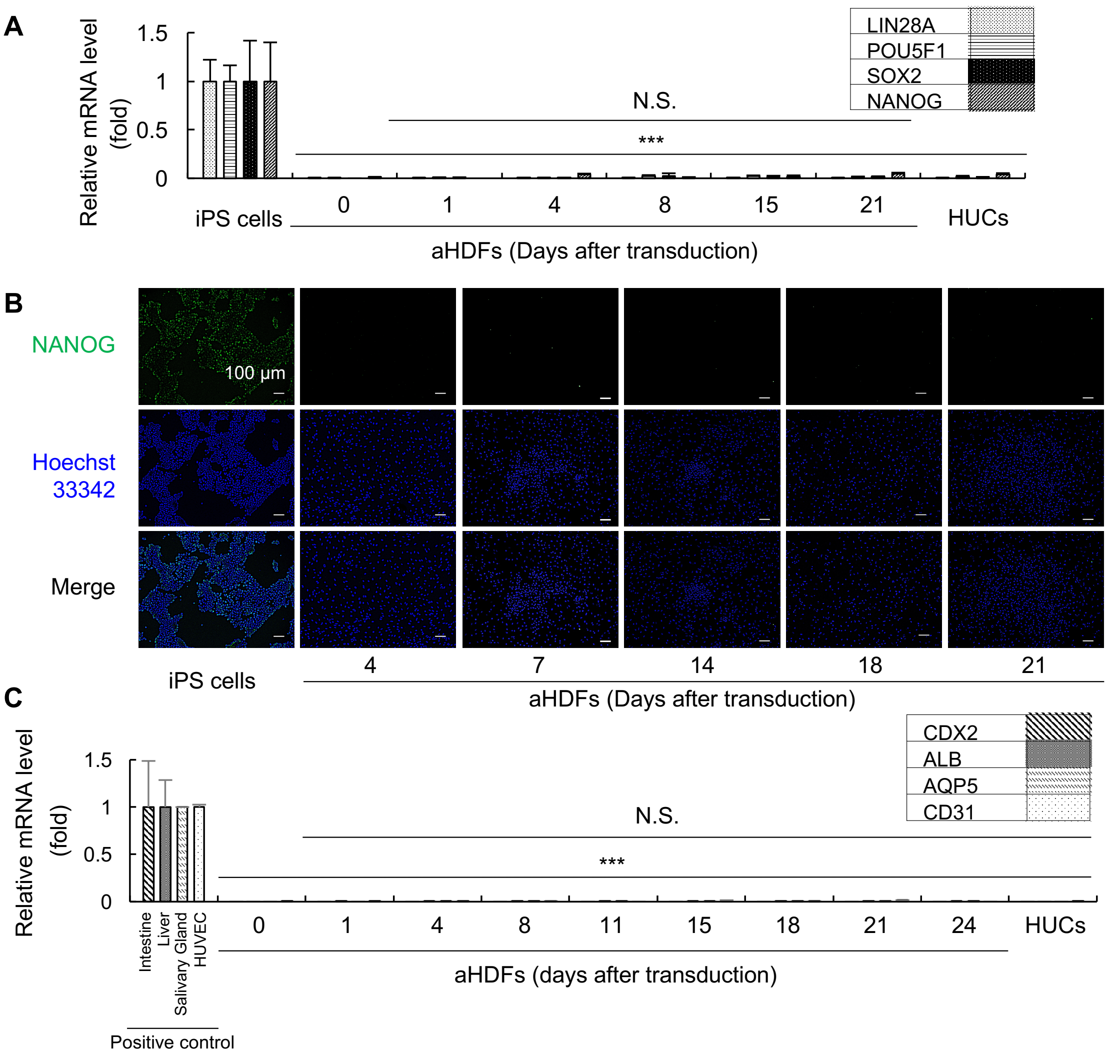

Supplementary Figure 7 | Pluripotent and some non-urothelial cell markers were not detected during the conversion from aHDFs into dUCs.

aHDFs were seeded in laminin-coated plates, transduced with FTLK, and cultured in Standard Medium (days 1 to 3) and CnT-Prime (days 4 to 21). (**A**) On the indicated days, RNA was extracted from the cells and human iPS cells. mRNA for the indicated genes were evaluated by real-time RT-PCR. mRNA from HUCs was also tested. Values (averages +/- SD) (n=3) were normalized to β-actin mRNA and expressed relative to values for iPS cells (set to 1.0). N.S.: not significant vs. Day 0. ***P<0.001 vs. iPS cells. (**B**) Cells were immunostained with anti-NANOG antibody, while cell nuclei were also

stained with Hoechst 33342. Human iPS cells were also stained as a control (Leftmost panels). (**C**) On the indicated days, RNA was extracted from the cells and mRNA for the indicated genes was evaluated by real-time RT-PCR. mRNA from human intestine, liver and HUCs was also tested. Values (averages +/- SD) (n=3) were normalized to β-actin mRNA and expressed relative to values for intestine (CDX2), liver (ALB), salivary gland (AQP5) and HUVEC (CD31) (set to 1.0). N.S.: not significant vs. Day 0. $^{***}P<0.001$ vs. positive control.

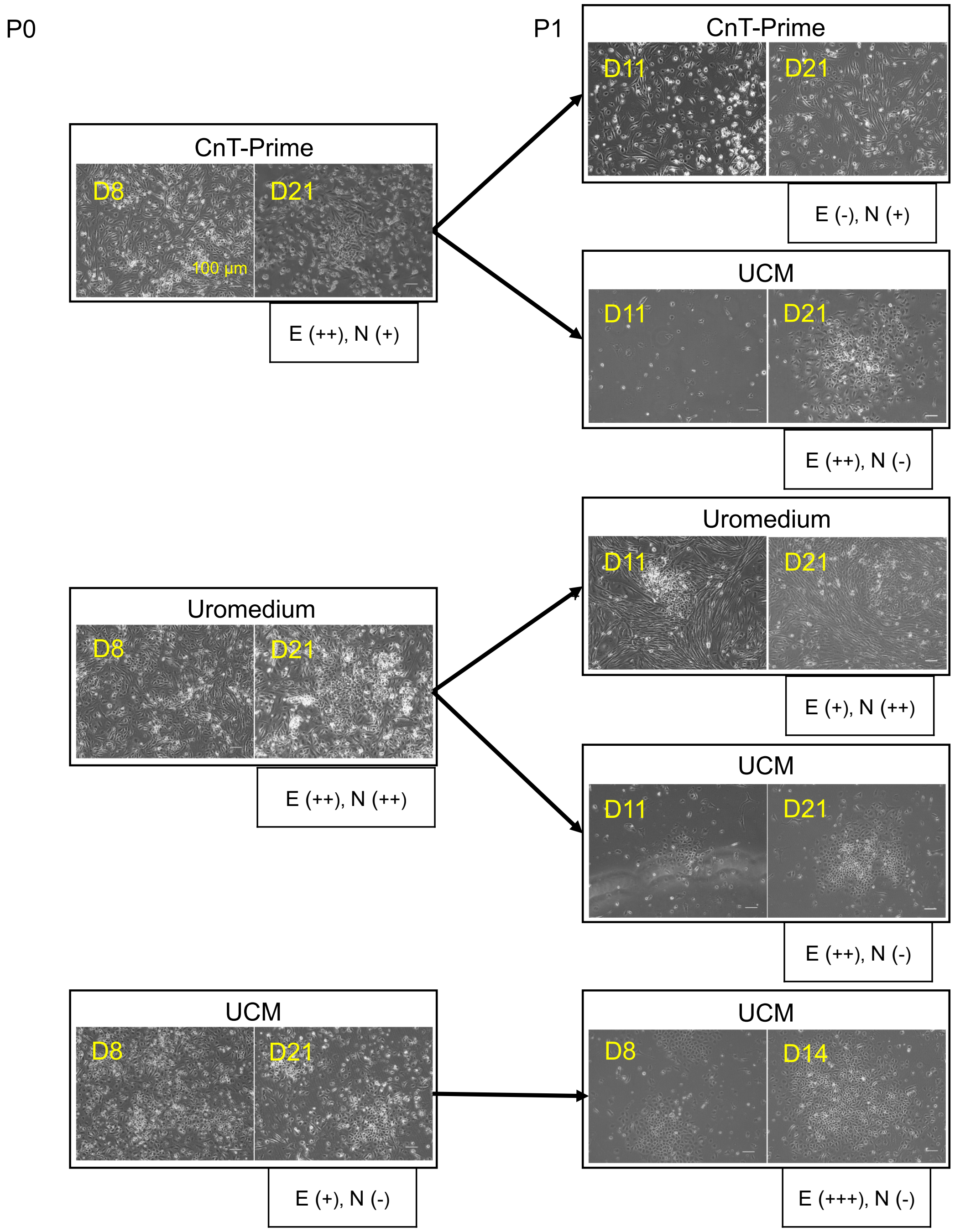

Supplementary Figure 8 | dUCs were successfully passaged depending on culture media.

FTLK-transduced aHDFs were cultured in CnT-Prime, "Uromedium" or "UCM" from day 4 to 21 to obtain P0 dUCs. They were detached from the culture plates, reseeded in new culture plates, and cultured in the indicated media for 14 or 21 days. Phase contrast

microscopic images of the cells are shown. E, Epithelial colony formation; N, Proliferation of non-epithelial cells.

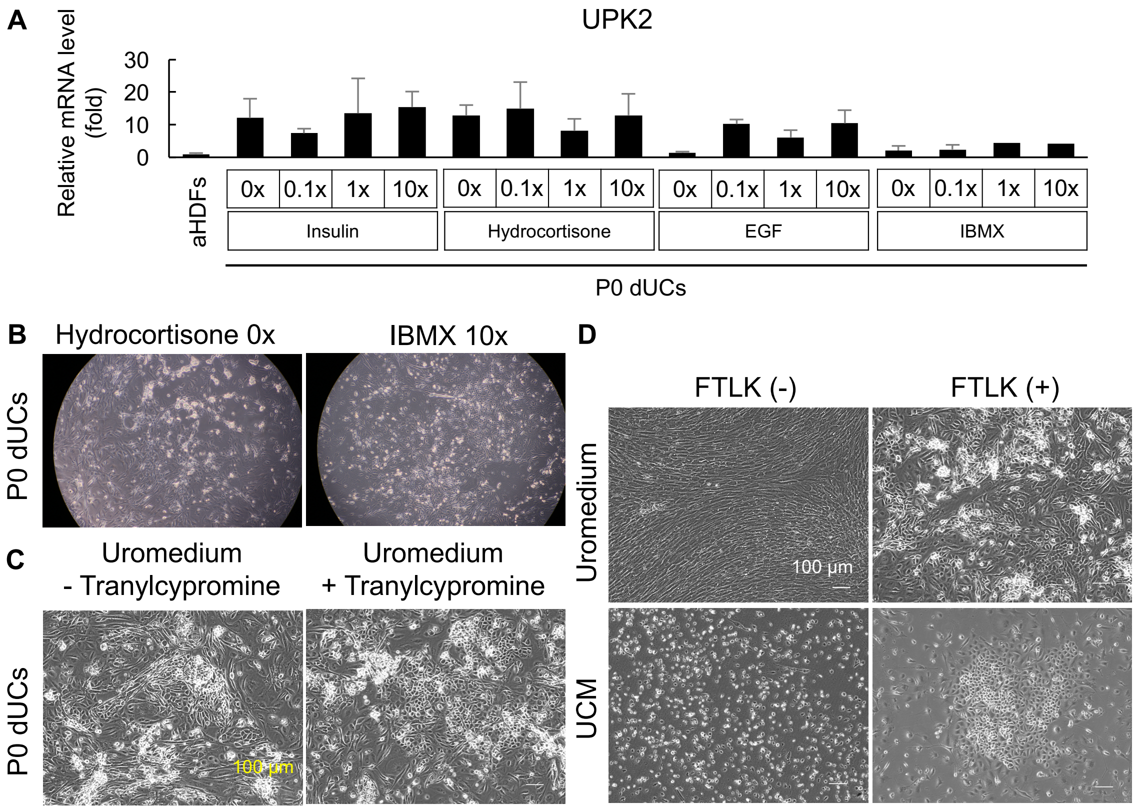

Supplementary Figure 9 | Determination of components of "UCM" adequate for selective expansion of dUCs.

(**A**) FTLK-transduced aHDFs were cultured in modified "Uromedium" with different concentrations of insulin, hydrocortisone, EGF and IBMX. Total RNA was extracted from the cells on day 21 as well as from non-transduced aHDFs as control, and UPK2 mRNA levels were evaluated by real-time RT-PCR. Values (averages +/- SD) (n=3) were normalized to β-actin mRNA and expressed relative to values for non-transduced aHDFs (set to 1.0). (**B**) Phase contrast microscopic images of FTLK-transduced aHDFs cultured for 21 days in modified "Uromedium" with x 0 hydrocortisone and x 10 IBMX. Original magnification: x 10. (**C**) FTLK-transduced aHDFs were cultured in "Uromedium" with or without supplementation of 1 μM tranylcypromine for 21 days. Phase contrast microscopic images of the cells are shown. (**D**) FTLK-transduced and non-transduced

aHDFs were cultured in “Uromedium” or “UCM” for 21 days. Phase contrast microscopic images of the cells are shown.

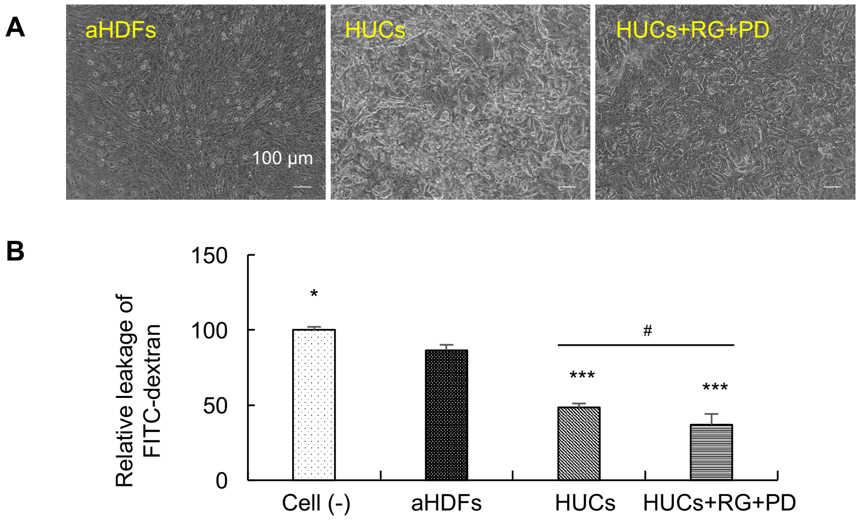

Supplementary Figure 10

HUCs showed significant barrier function in in vitro permeability assay.

HUCs resuspended in Urolife D Complete Medium (Urolife) and aHDFs were seeded into inner chambers of Transwell plates at 1 x 10^5 cells/well. Some aliquots of the HUCs were treated with 1μM each of a PPARγ activator (rosiglitazone; Sigma-Aldrich) and an EGFR inhibitor (PD153035; TCI) to induce terminal differentiation as previously described **(1)**. Four days later, confocal microscopic images of the inner chamber (**A**), while permeability assay was performed as described in the Materials and Methods (**B**). Values are mean +/- SD (n=3). $^{*}P<0.01$ and $^{***}P<0.001$ vs. aHDFs. $^{\#}P<0.05$ between groups.

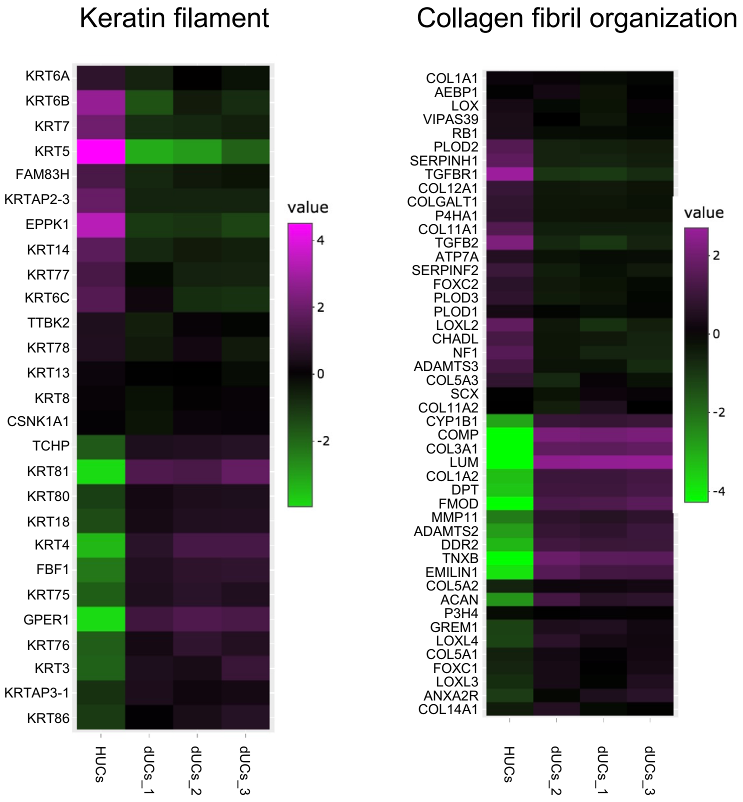

Supplementary Figure 11 | Gene Ontology analysis of dUCs and HUCs.

Total RNA of P2 dUCs (n=3) and HUCs (n=1) were sequenced with Illumina Novaseq 6000 (Illumina; paired-end, 150 bp) and analyzed by iDEP8.1 (2). Heatmaps for GO genesets comparing P2 dUCs with HUCs are shown.

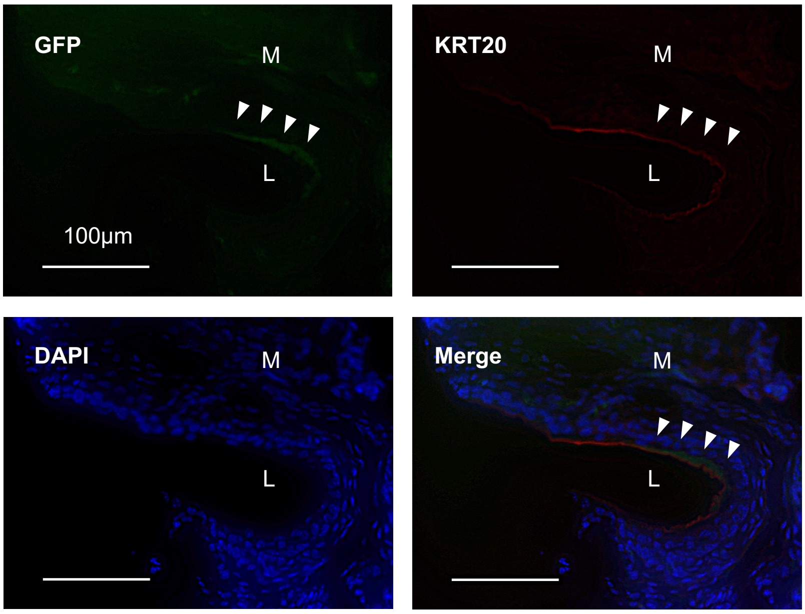

Supplementary Figure 12 | dUCs expressed a terminal differentiation marker at the urinary mucosa in urothelium-injured mouse bladders.

FTLKG-transduced aHDFs cultured for 4 days in Standard Medium were transplanted into urothelium-injured bladder of NOG/SCID mice as in Fig. 5c. One week after the transplantation, mice were sacrificed and urinary bladder tissue specimens were subjected to immunohistochemical analysis using anti-KRT20 antibody and nuclear staining with DAPI. M and L represent muscle and lumen of the bladder, respectively. Arrowheads represent GFP-labeled cells.

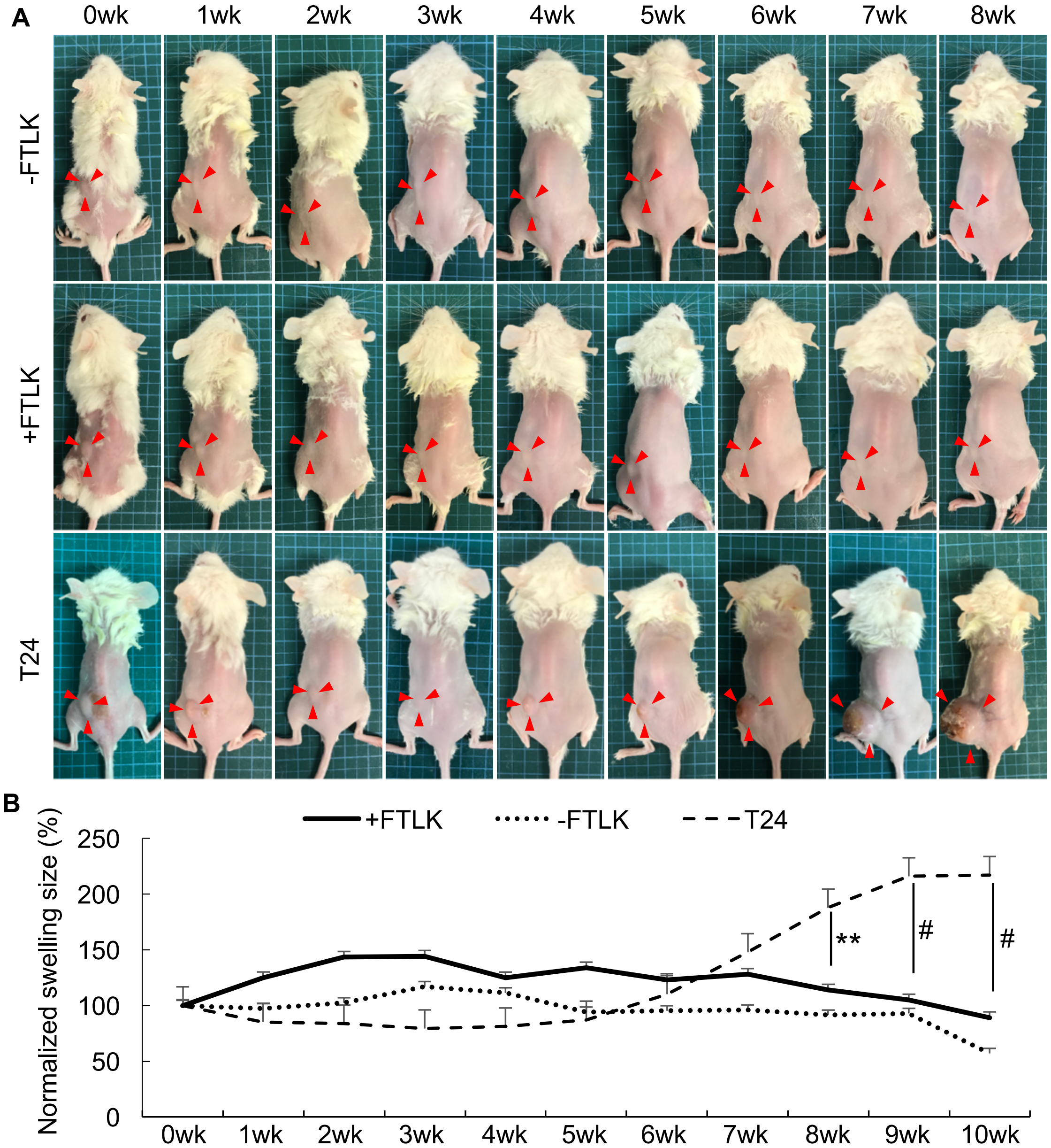

Supplementary Figure 13 | dUCs did not form tumor in vivo.

FTLK-transduced aHDFs (4 days after transduction) (+FTLK), non-transduced aHDFs (-FTLK) or T24 were mixed with Matrigel (Corning) and subcutaneously inoculated into NOG/SCID mice at a dose of 1 x 10^7 cells/100 µL/mouse (n=4 mice per each group). (**A**) Macroscopic images of the mice are shown. Red triangles indicate swelling subcutaneous tissues or tumors at the inoculation sites. (**B**) Volumes of the swelling subcutaneous

tissues or tumors were measured every week. Means +/- SD of the volume was normalized to the value at 0 wk (n=4). **P<0.01 vs. -FTLK, #P<0.001 vs. both -FTLK and +FTLK.

References

1. Varley, C. L. *et al.* Role of PPARgamma and EGFR signalling in the urothelial terminal differentiation programme. *J. Cell. Sci.* **117**, 2029-2036 (2004)
2. Ge, S. X., Son, E. W. & Yao, R. iDEP: an integrated web application for differential expression and pathway analysis of R NA-Seq data. *BMC Bioinformatics* **19**, 10.1186/s12859-018-2486-6 (2018).
